# Corneal Mucin‐Targeting Liposome Nanoplatforms Enable Effective Treatment of Dry Eye Diseases by Integrated Regulation of Ferroptosis and Inflammation

**DOI:** 10.1002/advs.202411172

**Published:** 2024-11-28

**Authors:** Yin Zhang, Tinglian Zhou, Kai Wang, Chenqi Luo, Dan Chen, Zeen Lv, Haijie Han, Ke Yao

**Affiliations:** ^1^ Eye Center the Second Affiliated Hospital School of Medicine Zhejiang University Zhejiang Provincial Key Laboratory of Ophthalmology Zhejiang Provincial Clinical Research Center for Eye Diseases Zhejiang Provincial Engineering Institute on Eye Diseases Hangzhou 310009 China

**Keywords:** cyclosporine A, dry eye disease, ferroptosis, ferrostatin‐1, oxidative stress

## Abstract

The incidence of dry eye disease (DED) has been increasing annually worldwide, creating an urgent need for new therapies. Due to the multifactorial mechanism underlying DED, traditional medications focused on decreasing ocular surface inflammation have been unable to address all the harmful factors and fail to achieve a complete clinical cure. Ferroptosis, a new form of programmed cell death characterized by lipid peroxidation, has become a pivotal contributor to dry eye oxidative stress‐driven pathology. Therefore, therapeutic targeting of ferroptosis may be an attractive option for dry eye management. Herein, a sialic acid‐targeting peptide‐modified liposome loaded with Cyclosporine A (CsA), a typical anti‐inflammatory drug, and Ferrostatin‐1 (Fer‐1), a selective ferroptosis inhibitor, is developed termed as CF@SNPs, for combing and sustaining DED treatment. This multifunctional liposomal encapsulation demonstrates excellent aqueous solubility; moreover, the sialic acid‐targeting peptide prolongs ocular surface retention, further enhancing therapeutic efficacy. The CF@SNPs treatment comprehensively alleviates DED symptoms, including improving corneal defects, augmenting goblet cell count, and restoring tear secretion. Specifically, CF@SNPs attenuate dry eye pathology by suppressing p53‐SLC7A11‐GSH‐dependent ferroptosis and TNF‐α‐associated inflammatory cascades, accompanied by favorable biocompatibility in vivo. These results underscore the promising potential of this superior nano‐formulation for DED pharmacotherapy.

## Graphical abstract

A sialic acid‐targeting peptide‐modified liposome loaded with Cyclosporine A and Ferrostatin‐1, termed CF@SNPs, is developed for combating DED. The CF@SNPs comprehensively alleviated DED symptoms, including improving corneal defects, augmenting goblet cell count, and restoring tear secretion. Specifically, CF@SNPs attenuated dry eye pathology by suppressing p53‐SLC7A11‐GSH‐dependent ferroptosis and TNF‐α‐associated inflammatory cascades, accompanied by favorable biocompatibility.

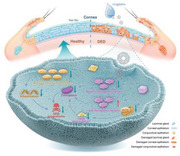

## 1 Introduction

Dry eye disease (DED) is a multifactorial disorder characterized by disruption of tear film homeostasis, with an estimated prevalence of 5% to 50% worldwide.^[^ ^1^ ^]^ Although DED is mainly associated with age, frequent screen use and wearing contact lenses lead to increasing diagnoses in younger people.^[^ ^2^, ^3^ ^]^ Irritation, burning, mild to severe ocular inflammation, and itching eye fatigue are all characterizations of DED, which may cause potential damage to the conjunctiva, cornea, and even blindness.^[^ ^4^, ^5^ ^]^ The impact on visual acuity and life quality demands attention, highlighting the pressing need for effectual prevention and treatment for DED.

Ocular inflammation and hyperosmolarity of tear film are considered the hallmarks of DED.^[^ ^6^, ^7^ ^]^ Diminished tear secretion or dryness exerts pressure stimuli on the ocular surface, elevating tear film osmolarity and depriving the cornea of essential nutrients. Moreover, these initiation factors trigger the secretion of pro‐inflammatory cytokines, matrix metalloproteinases, and chemokines, and these inflammatory mediators mutually regulate and enhance each other, amplifying the inflammatory cascade that undermines the integrity of the corneal barrier.^[^ ^8^ ^]^ Consequently, this cascade of events disrupts tear film stability, perpetuating ocular surface hyperosmolarity, reducing tear secretion, and trapping dry eye in a vicious cycle of dysfunction. As such, inflammation has been a primary target for treating DED over the past decades. Cyclosporine A (CsA), an FDA‐approved anti‐inflammatory medication for DED,^[^ ^9^, ^10^ ^]^ has demonstrated significant efficacy in clinical, marking a notable advancement in treatment. However, CsA does not address the acute effects of dryness on the ocular surface, and prolonged medication usage may lead to unexpected side effects.^[^ ^11^, ^12^ ^]^ These clinical issues give rise to the hypothesis that inflammation may not be prominent in many unresponsive cases or the presence of additional factors might diminish its dominant role.

Alternatively, it has been reported that hyperosmolarity of tear film can trigger oxidative stress pathways.^[^ ^13^ ^]^ Elevated oxidative stress levels have been observed in DED patients' tear film and ocular surface, correlating with various clinical indicators of ocular surface function. Ferroptosis, a newly identified form of non‐apoptotic cell death,^[^ ^14^, ^15^ ^]^ is characterized by elevated levels of iron‐dependent lipid peroxidation and reactive oxygen species (ROS),^[^ ^16^, ^17^, ^18^, ^19^ ^]^ and the progression can selectively be recovered by ferrostatin‐1 (Fer‐1), a scavenger of lipid peroxidation.^[^ ^20^ ^]^ Previous investigations have highlighted the pivotal regulatory role of ferroptosis in various corneal pathophysiological processes.^[^ ^21^, ^22^ ^]^ Recently, ferroptosis was observed in the progression of DED,^[^ ^23^ ^]^ suggesting the therapeutic promise via targeting ferroptosis for this ocular surface disorder. Nonetheless, the therapeutic effects and underlying mechanisms of targeting ferroptosis at the onset of DED warrant us to investigate further.

Traditional eye drops for DED were reported to have demonstrated limitations like poor drug availability and side effects upon frequent administration,^[^ ^24^, ^25^ ^]^ prompting the development of formulas that can improve drug retention and enable precision targeting for better treatment outcomes. Salivary acid, abundant in ocular epithelial cells, serves as a potential anchoring point for ocular surface tissues,^[^ ^26^, ^27^ ^]^ providing a rationale for our investigation of novel therapy for DED. In this study, we aimed to uncover the regulated role of ferroptosis underlying DED pathogenesis. On this basis, mucoadhesion liposomes co‐delivering CsA and Fer‐1 to achieve synergistic anti‐inflammatory and anti‐ferroptosis effects on the ocular surface. Technologically, we engineered the liposome surface by targeting peptides containing sialic acid, which is abundant in ocular epithelial cells and serves as a potential anchoring point for ocular surface tissues to control the release activity through ligand‐receptor interactions. The mechanistic study revealed that our multifunctional nanoplatforms demonstrated a superior therapeutic effect by inhibiting p53‐SLC7A11‐GSH‐dependent ferroptosis and TNF‐α associated inflammatory cascades. This approach offers a more precise, comprehensive, and effective treatment strategy for DED management.

## 2 Results

### 2.1 DED Generates Corneal Injury Through Ferroptosis

**Figure 1:**
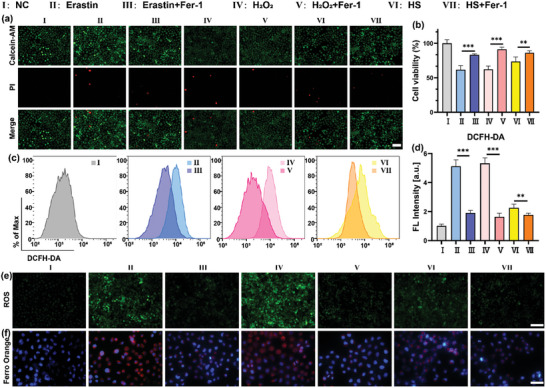
Cytoprotective effects, anti‐ROS, and anti‐iron accumulation properties of Fer‐1 in HCECs were assessed. a) Representative Live/Dead images depict HCECs following various treatments, with green indicating live cells and red indicating dead cells. The scale bar represents 100 µm. b) Cell viability of HCECs assessed via CCK‐8 assay. Express data as mean ± standard deviation (SD). n = 3. ^**^ *P* < 0.01, ^***^ *P* < 0.001. c) Flow cytometry analysis and d) quantitative results illustrate ROS levels in HCECs following various therapies. Present data as mean ± SD. n = 3. ^**^ *P* < 0.01, ^***^ *P* < 0.001. e) Representative images demonstrate ROS levels visualized through DCFH‐DA staining (green). The scale bar represents 100 µm. f) Representative images display ferric ion levels visualized by Ferro Orange (red), with nuclei stained by DAPI (blue). The scale bar represents 50 µm.

To investigate the potential involvement of ferroptosis in the pathogenesis of DED, we initially established in vitro DED models using human corneal epithelial cells (HCECs) subjected to hydrogen peroxide (H_2_O_2_) and hyperosmotic stress (HS) treatments. Subsequently, we conducted rescue experiments targeting cell death using the ferroptosis inhibitor Fer‐1. For comparative analysis, we employed the ferroptosis inducer Erastin as a positive control to induce cell death via ferroptosis. Cell viability in each treatment group was primarily assessed through live/dead staining (**Figure** **1**a) and CCK‐8 assay (Figure 1b). Our findings revealed that both the in vitro DED models and Erastin exposure elicited varying degrees of cell death in HCECs, whereas Fer‐1 intervention notably enhanced cell survival rates. Among them, the cell survival rate was increased by 21.37%, 29.11%, and 12.83% in Erastin, H_2_O_2_, and HS injury, respectively.

Oxidative stress stands as a pivotal factor driving injury in DED.^[^ ^28^, ^29^, ^30^ ^]^ To assess the therapeutic efficacy of ferroptosis inhibition, we quantified intracellular levels of ROS. After induction by a 2,7‐dichlorofluorescein (DCFH‐DA) fluorescent probe, flow cytometry (Figure 1c,d) and fluorescence staining (Figures 1e and S1, Supporting Information) were used to determine the results. Our findings indicated that all Erastin induction, H_2_O_2_ induction, and HS induction show a significant elevation in intracellular ROS levels compared to the control group, and this improvement could be restrained to varying degrees under Fer‐1 intervention. These observations collectively underscore the potential of Fer‐1 administration in ameliorating corneal damage associated with in vitro dry eye conditions.

Lipid peroxidation and iron accumulation are pivotal determinants in the onset of ferroptosis.^[^ ^31^, ^32^, ^33^ ^]^ To further investigate the presence of ferroptosis in vitro DED models, we conducted assessments of intracellular iron ion and lipid peroxidation levels. Ferro Orange staining (Figure 1f) revealed a marked increase in intracellular iron ion content in both Erastin‐induced ferroptosis and in models of DED induced by H_2_O_2_ and HS conditions, compared to the control group. Remarkably, treatment with the Fer‐1 substantially attenuated the accumulation of intracellular iron ions. In H_2_O_2_ or HS environments, the fluorescence intensity of ferrous ions could be reduced by ≈20% after treatment with Fer‐1 (Figure S2, Supporting Information).

**Figure 2:**
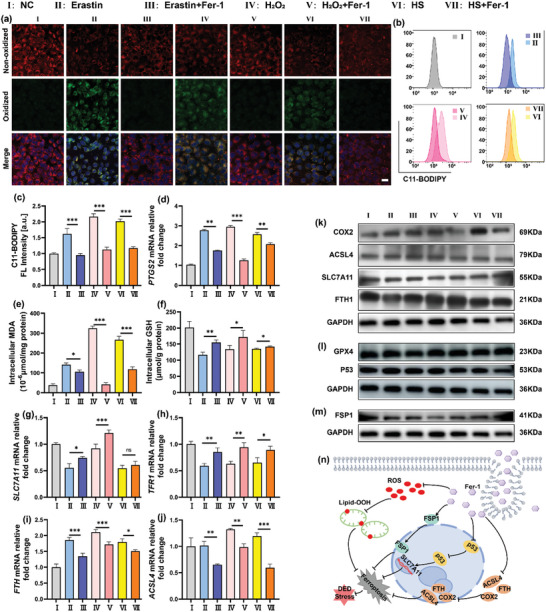
Anti‐lipid peroxidation and anti‐ferroptosis effects of Fer‐1 in HCECs. a) Representative fluorescent images of lipid peroxidation, as assessed by C11‐BODIPY staining, illustrated non‐oxidized lipids in red and oxidized lipids in green, with nuclei stained by DAPI (blue). The scale bar represents 20 µm. b) Flow cytometry analysis and c) the corresponding quantitative lipid peroxidation results in HCECs subjected to various therapies. Present data as mean ± SD. n = 3. ^***^ *P* < 0.001. d) Relative fold change in *PTGS2* mRNA expression in HCECs following various treatments. Present data as mean ± SD. n = 3. ^**^ *P* < 0.01, ^***^ *P* < 0.001. e) Quantification of malondialdehyde (MDA) levels in HCECs. Present data as mean ± SD. n = 3. ^*^ *P* < 0.05, ^***^ *P* < 0.001. f) Glutathione (GSH) levels in HCECs. Present data as mean ± SD. n = 3. ^*^ *P* < 0.05. ^**^ *P* < 0.01. g‐j) Expression of ferroptosis‐related genes. Present data as mean ± SD. n = 3. ^*^ *P* < 0.05, ^**^ *P* < 0.01, ^***^ *P* < 0.001. k‐m) Protein levels of COX2, ACSL4, SLC7A11, GPX4, FTH1 and GPX4, p53, FSP1. n) Schematic diagram of the proposed mechanism of the inhibition of intracellular signaling pathways and Fer‐1 action targets.

Lipid peroxidation stands as a critical hallmark of ferroptosis. Accordingly, we evaluated parameters associated with lipid peroxidation. Initially, staining with the C11‐BODIPY fluorescent probe for lipid peroxidation (**Figures** **2**a and S3, Supporting Information) alongside flow cytometry results (Figure 2b,c) demonstrated a notable increase in intracellular lipid peroxidation levels following Erastin, H_2_O_2_, and HS treatments, with varying degrees of reduction observed upon Fer‐1 treatment. Additionally, *PTGS2* emerges as a pivotal marker gene for lipid peroxidation,^[^ ^34^ ^]^ with alterations in its expression commonly serving as a classical indicator of ferroptosis onset and severity. Notably, we observed significant upregulation of *PTGS2* mRNA expression levels post Erastin, H_2_O_2_, and HS treatments, with substantial downregulation evident upon Fer‐1 intervention (Figure 2d). Furthermore, we assessed the protein levels of malondialdehyde (MDA), the final product of lipid peroxidation, revealing that Fer‐1 treatment notably ameliorated the heightened lipid peroxidation induced by H_2_O_2_ and HS (Figure 2e). Glutathione (GSH) is a pivotal antioxidant and a negative ferroptosis regulator.^[^ ^35^ ^]^ Our investigation revealed that DED conditions in vitro led to a depletion of intracellular GSH levels. Remarkably, this consumption could be effectively restored by the Fer‐1 administration (Figure 2f). In conclusion, these findings preliminarily suggest a crucial role of ferroptosis in the in vitro DED model, and treating with Fer‐1 could improve ferroptosis more or less.

Subsequently, to further illustrate the role of ferroptosis in the DED model, we investigated the expression of genes associated with ferroptosis. Our findings revealed that upon induction by Erastin, H_2_O_2_, and HS, there was a significant reduction in the expressions of *SLC7A11* (Figure 2g) and *TFRC* (Figure 2h). In contrast, the expressions of *FTH* (Figure 2i) and *ACSL4* (Figure 2j) were significantly increased. Remarkably, Fer‐1 treatment markedly ameliorated these alterations. Western blot analysis (Figure 2k) further corroborated the mRNA‐level results. Given the significant changes observed in both mRNA and protein levels of SLC7A11, we speculated on the involvement of the SLC7A11 signaling pathway. Consequently, we measured the protein level of p53 (Figure 2l), an upstream regulator of SLC7A11,^[^ ^36^ ^]^ revealing a substantial increase in its expression following induction by H_2_O_2_ and HS. Notably, Fer‐1 treatment mitigated this increase, indicating a potential role of H_2_O_2_ and HS in inducing ferroptosis via the p53‐SLC7A11 pathway. However, upon induction by Erastin, we observed a significant reduction in the protein level of GPX4, and Fer‐1 could rescue this reduction. Conversely, induction by H_2_O_2_ and HS did not result in a substantial decrease in GPX4 level. This finding suggests that H_2_O_2_ and HS‐induced ferroptosis may not be primarily mediated by GPX4 (Figure 2l). And then, we estimated the protein level of ferroptosis suppressor protein1 (FSP1), a glutathione‐independent ferroptosis suppressor.^[^ ^37^, ^38^ ^]^ Our findings (Figure 2m) revealed that upon induction by Erastin, H_2_O_2_, and HS, there was a significant reduction in the expressions of FSP1, while Fer‐1 markedly ameliorated this outcome.

Inflammation assumes a central role in the pathogenesis of DED, exerting a profound influence on its vicious cycle.^[^ ^39^ ^]^ Thus, it is imperative to assess indicators associated with inflammation. Examination of inflammatory outcomes (Figures S4, S5, S6, and S7, Supporting Information) revealed a marked increase in the fluorescence intensity of vital inflammatory mediators such as IL‐1β, TNF‐α, and MMP‐9 following induction with Erastin, H_2_O_2_, and HS. Importantly, intervention with Fer‐1 exhibited a certain degree of amelioration in these alterations. Meanwhile, the mRNA expression profiles (Figures S8,S9,S10, Supporting Information) harmonized with the findings from fluorescence staining assays. As shown in the devised mechanism (Figure 2n), H_2_O_2_ and HS possibly accelerated DED deterioration by promoting ferroptosis and upregulating downstream p53‐SLC7A11 activation while Fer‐1 could improve the condition by downgrading the corresponding pathway.

### 2.2 Preparation and Characterization of Anti‐Ferroptosis and Anti‐Inflammatory Co‐Loaded Liposomes Targeted to the Ocular Surface

**Figure 3:**
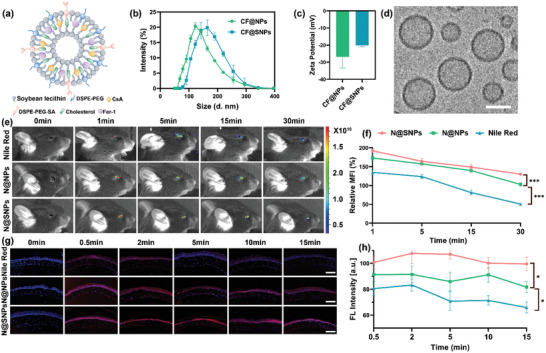
Characterization and preocular retention assessment of CF@SNPs. a) Schematic representation of CF@SNPs. DLS measured b) D*_h_* and c) zeta potential of CF@SNPs and CF@NPs. d) Cryo‐TEM image of CF@SNPs, with the scale bar representing 100 nm. Real‐time, in vivo e) fluorescence imaging and f) quantitative analysis of fluorescence intensity changes of free Nile Red (99 µM) compared to N@NPs and N@SNPs (with Nile Red concentration equivalent to 99 µM) on the anesthetized mice eyes at individual time points (0, 1, 5, 15, and 30 min). n = 3. ^***^ *P* < 0.001. g) Fluorescence images and h) quantitative analysis of average fluorescence intensity of ocular corneal frozen sections after administration with Nile Red or N@NPs, N@SNPs on conscious mice for 0, 0.5, 2, 5, 10, and 15 min. The scale bar is 20 µm. Data are presented as mean ±SD. n = 3. ^*^ *P* < 0.05.

While Fer‐1 treatment exhibits some efficacy in ameliorating the corneal damage induced by DED, its therapeutic potential remains constrained. Furthermore, for in vivo applications, its limited water solubility and rapid dissipation lead to diminished bioavailability. To address these challenges, we developed a sialic acid targeting peptides modified and Fer‐1 and CsA co‐loaded liposomes via membrane hydration, denoted as CF@SNPs, as depicted in **Figure** **3**a. Analysis of dynamic light scattering (DLS) analysis revealed that the intensity‐average hydrodynamic diameter (D*_h_*) of the CF@SNPs and CF@NPs (liposomes co‐encapsulated CsA and Fer‐1) were 153.4 ± 43.7 nm and 122.2 ± 21.1 nm, respectively (Figures 3b). Zeta potentials were ‐20.2 ± 5.33 mV and ‐26.9 ± 6.56 mV, respectively (Figure 3c). Cryogenic transmission electron microscopy (cryo‐TEM, FEI) imaging (Figure 3d) further elucidated the morphology of CF@SNPs, exhibiting a distinct spherical shape and individual particle dispersion. Notably, the size of CF@SNPs determined via cryo‐TEM was 145.8 ± 22.94 nm, smaller than the D*_h_* obtained through DLS measurements. This variance may be attributed to the hydration or the extensive solvation of nanoparticles during DLS analysis.

Prolonging ocular surface residence time represents a critical strategy for enhancing drug bioavailability. Results from in vivo fluorescence imaging (Figures 3e) and fluorescence intensity assessments upon frozen sectioning (Figures 3f) indicate that, over time, the ocular retention of N@SNPs (Nile Red encapsulated within sialic acid targeting peptide‐modified liposome) and N@NPs (Nile Red encapsulated within liposome) is markedly superior to that of the dissociative Nile Red solution. In addition, the retention duration of N@SNPs surpasses that of N@NPs. Specifically, at the 1‐min interval, the residual fluorescence intensity (Figure 3g) in anesthetized mice administered N@SNPs remained notably high at 192.22 ± 5.27 compared to a mere 134.89 ± 6.34 in mice administered free Nile Red. At the 30 min interval, the residual fluorescence intensity in anesthetized mice administered N@SNPs was 2.6 times that of mice administered free Nile Red. Similarly, in conscious mice subjected to eye drop administration for 15 min, fluorescence intensity (Figure 3h) measurements obtained from eye slices revealed values of 99.50 ± 5.21 for N@SNPs, 81.68 ± 4.36 for N@NPs, and only 65.98 ± 4.29 for free Nile Red.

### 2.3 Biocompatibility Evaluation of CF@SNPs

Normal mice were stimulated with various drugs, and then the corneas were examined by the slit‐lamp under white light and cobalt blue light to monitor the ocular surface situation. Images of the anterior (Figure S11a, Supporting Information) and lateral (Figure S11b, Supporting Information) mine corneas exhibited intact corneal integrity under white light. Fluorescein sodium staining under cobalt blue light (Figure S11c, Supporting Information) revealed no discernible fluorescence sodium staining in any group. Similarly, histological examination of corneal sections stained with hematoxylin and eosin (H&E) demonstrated no significant pathological alterations in the corneas of treated mice compared to normal mice (Figure S11d, Supporting Information).

Furthermore, in the following administration of eye drops containing various drugs and subsequent organ removal, including the heart, kidneys, lungs, liver, and spleen, H&E staining images (Figure S12, Supporting Information) exhibited no appreciable differences in organ morphology between treated and normal mice. No evident lesions were observed in any of the examined organs.

Calcein‐AM/PI staining (Figures S13 and S14, Supporting Information) and CCK‐8 assays (Figures S15 and S16, Supporting Information) were employed to assess the cytotoxicity of CF@SNPs on corneal epithelial cells and macrophages, respectively. Following CF@SNPs treatment, only green fluorescence indicative of live cells was observed, with no red fluorescence denoting cell death. Additionally, CCK‐8 assays revealed that the cell survival rate remained exceeded 90% actually at a concentration of 20 µM.

### 2.4 The Therapeutic Effect of Ocular Surface Targeted Co‐Loaded Liposomes on Corneal Injury Caused by DED

**Figure 4:**
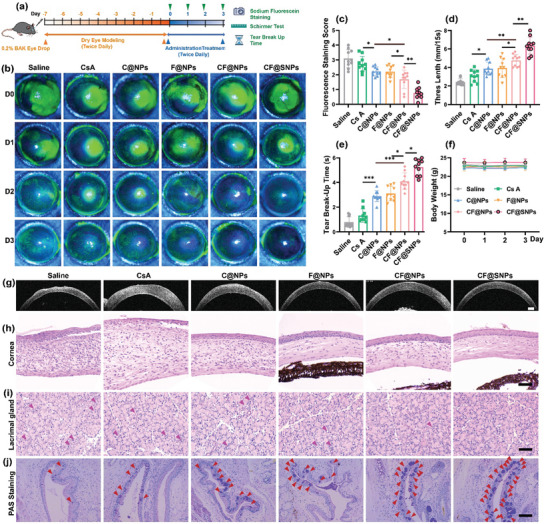
The therapeutic effects of miscellaneous treatments on DED mice induced by BAK were assessed. a) Schematic of DED model establishment and treatment protocol. b) Representative images of fluorescein staining and suiting staining scores, d) wetted phenol thread lengths, and e) tear breakup time (TBUT) in DED mice after different treatments over three days. f) Daily body weight records of DED mice with various treatments. Data presented as mean ± SD. n = 10. ^*^ *P* < 0.05, ^**^ *P* < 0.01, ^***^ *P* < 0.001. Representative images of g) anterior segment OCT, h) cornea, i) lacrimal gland, and j) conjunctival epithelium stained with periodic acid‐Schiff (PAS). Carmine triangle: lacrimal gland tissue structural disorder and lymphocyte infiltration. Red triangle: goblet cells. Scale bar: 100 µm.

Following the in vivo DED model established, as detailed in **Figure** **4**a, various treatments were administered to assess clinical efficacy over days 0, 1, 2, and 3 post treatments. After fluorescein sodium staining, corneal images were captured using a slit lamp under cobalt blue light (Figure 4b), followed by intensity scoring (Figures 4c and S17, Supporting Information). Tear volume was evaluated utilizing phenol red cotton thread (Figures 4d and S18, Supporting Information), tear break‐up time (TBUT) was quantified (Figures 4e and S19, Supporting Information), and mouse weight was monitored. The mouse weight (Figure 4f) remained stable throughout the study period. On day 0, each group exhibited substantial positive staining, scoring 3.60 ± 0.367. Within 15 s, phenol red cotton thread measured only 2.79 ± 0.733 mm, with tear film rupture time at 0.180 ± 0.110 s, confirming successful DED model establishment. Subsequent drug administration twice daily led to varied improvements in clinical efficacy indicators. After three days, significant positive staining persisted in the free CsA group, scoring 2.73 ± 0.519. The CsA liposomes (C@NPs) group showed no significant discrepancy in positive spots comparison with the free CsA group, with a staining score reduced to 2.23 ± 0.306, phenol red cotton thread length of 3.88 ± 0.712 mm, and TUBT of 2.89 ± 0.436 s. Similarly, the Fer‐1 liposomes (F@NPs) group exhibited comparable results to C@NPs, scoring 2.21 ± 0.396, with phenol red cotton thread length of 3.95 ± 1.04 mm and TUBT of 3.11 ± 0.578 s. Moreover, combination therapy using CF@NPs significantly reduced fluorescein sodium positive spots, scoring 1.69 ± 0.632, with phenol red cotton thread length of 4.78 ± 0.646 mm and TUBT of 4.10 ± 0.667 s, demonstrating superior efficacy compared to C@NPs and F@NPs. Noteworthy, after CF@SNPs administration, almost no positive spots were observed in fluorescein sodium staining, scoring 0.760 ± 0.384, with phenol red cotton thread length of 6.37 ± 0.918 mm and TUBT of 5.25 ± 0.950 s, indicating significantly enhanced efficacy compared to CF@NPs alone.

Three days after administration, corneal injury was evaluated using optical coherence tomography (OCT) imaging. As shown in Figure 4g, significant corneal injury and reduction of corneal smoothness were found in the saline group, with noticeable thickening and corneal smoothness reduction observed in the CsA group, indicating sustained injury. However, the C@NPs and F@NPs groups displayed some improvement in corneal injury and corneal smoothness. Remarkably, CF@NPs treatment eliminated corneal injury, while CF@SNPs not only resolved corneal damage but also showed no discernible difference in anterior segment OCT imaging compared to the normal group (Figure S20, Supporting Information).

We conducted histological assessments to analyze structural changes in the ocular surface post‐treatment. The corneal structure and cellular morphology were detected by H&E staining. Normal corneal cells exhibited dense, orderly arrangements without inflammatory cell infiltration (Figure S11, Supporting Information). Conversely, DED modeling resulted in superficial epithelial layer damage, visible thinning, reduced epithelial cell counts, and irregular morphology (Figure 4h). Additionally, the corneal stromal layer displayed increased thickness, a looser structure, excessive vacuoles, and inflammatory cell infiltration, consistent with previous findings. Following treatment, varying degrees of improvement in corneal structure and cellular morphology were observed. The CsA group exhibited minimal improved superficial layer damage and inflammatory cell infiltration but significant corneal stromal thickening. Both C@NPs and F@NPs groups demonstrated intact, notably thickened superficial and upper corneal layers, reduced stromal thickness, and fewer inflammatory cells. CF@NPs treatment increased epithelial cell numbers and regular morphology compared to the F@NPs group. In the CF@SNPs group, the corneal epithelial layer remained intact with regular morphology, while the stromal layer appeared tight, with minimal vacuoles or inflammatory cells, similar to the healthy group.

The integrity of the tear film is vital for ocular surface health, composed of an aqueous layer, mucin layer, and lipid layer.^[^ ^40^ ^]^ Disruptions to this balance can result in tear film instability. The aqueous layer, predominantly produced by the lacrimal gland, constitutes the most abundant component. Comprised mainly of acinar cells (80%) and infiltrating immune cells (20%) such as lymphocytes, the gland typically shows uniform and well‐arranged acini in a healthy state.^[^ ^41^ ^]^ However, in DED models, the lacrimal gland structure undergoes significant disorganization, characterized by irregular acini size, disrupted arrangement, atrophy, fusion, intracellular vacuoles, and external lymphocyte infiltration (Figure 4i). The administration of CF@NPs and CF@SNPs significantly ameliorates the structural disruption associated with DED, approximating a healthy tear gland. Remarkably, CF@SNPs show pronounced efficacy in addressing acinar fusion.

Mucin, the main ingredient of the mucous layer, is predominantly produced by goblet cells, and its absence is a hallmark of DED.^[^ ^42^ ^]^ After DED modeling, periodic acid Schiff (PAS) glycogen staining results (Figure 4j) indicate goblet cell atrophy and a significant decrease in number in the saline group. Different treatments lead to varied increases in goblet cell numbers, with CF@NPs and CF@SNPs showing significantly better effects than other groups. Furthermore, CF@SNPs promote goblet cell proliferation with regular and round morphology.

### 2.5 The Therapeutic Effect of Ocular Surface Targeted Co‐Loaded Liposomes on Ferroptosis and Inflammation in DED

**Figure 5:**
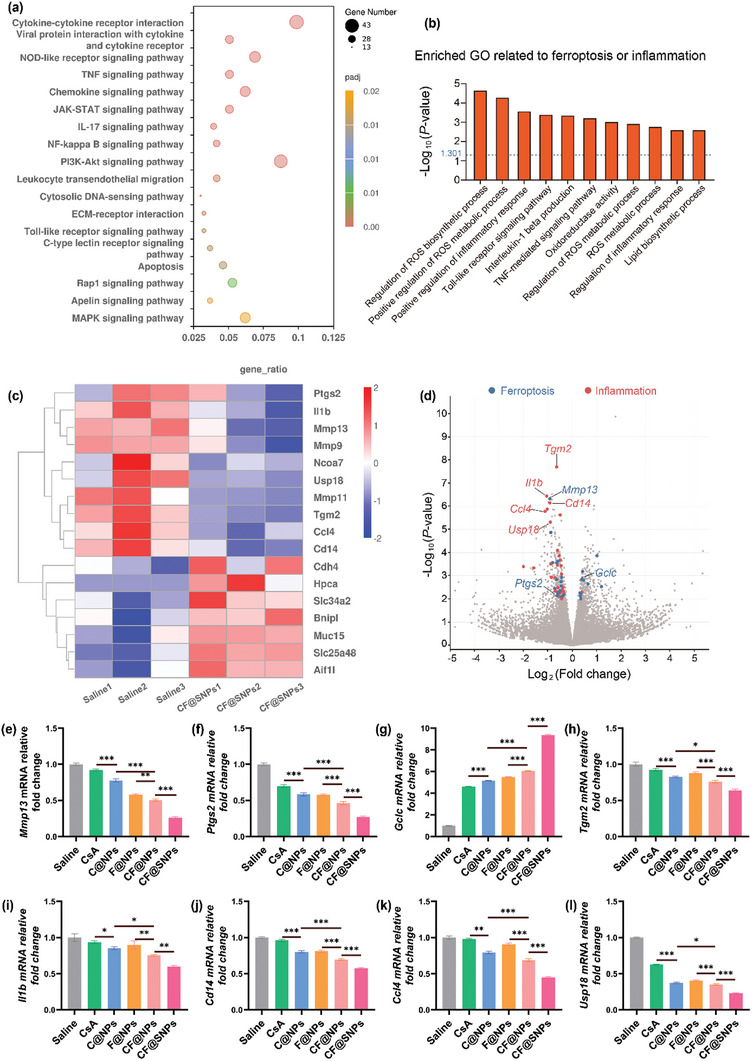
Anti‐inflammatory and anti‐ferroptosis effects of CF@SNPs in DED mice RNA‐seq analyses. a) CF@SNPs‐associated DEAS events functional analyses in DED mice by KEGG. b) Enriched GO related to ferroptosis and inflammation. c) Displayed markedly down‐regulated genes in DED mice after CF@SNPs treatment by heat map (fold change ≥ 1.5 and *P* < 0.05). d) Differential expression analysis. (e–l) Relative fold change in *Mmp13*, *Ptgs2*, *Gclc* and *Tgm2*, *Il1b*, *Cd14*, *Ccl4*, *Usp18* mRNA expression in DED mice following various treatments. n = 3. ^*^ *P* < 0.05. ^**^ *P* < 0.01. ^***^ *P* < 0.001.

To elucidate the therapeutic mechanisms of CF@SNPs, we conducted RNA‐seq analyses to delineate changes in gene expression between the DED model (saline‐treated group) and the CF@SNPs‐treated group. In light of our in vitro results, we scrutinized the impact of CF@SNPs treatment on genes implicated in ferroptosis and inflammation. We used the Kyoto Encyclopedia of Genes and Genomes (KEGG) to assess the corresponding gene pathway enrichment (**Figure** **5**a). Gene Ontology (GO) analysis of differentially expressed alternative splicing (DEAS) with the most observably concentrated pathways is shown in Figure 5b. Moreover, differential expression analysis (Figure 5c,d) revealed significant reductions in the expression of *Mmp13*, a ferroptosis‐associated marker, and *Ptgs2*, indicative of lipid peroxidation. Conversely, there was a noteworthy elevation in the expression of *Gclc*, which was pivotal in suppressing ferroptosis via GSH synthesis. Additionally, the expression levels of inflammatory markers, including *Tgm2*, *Il1b*, *Cd14*, *Ccl4*, and *Usp18*, exhibited marked increases. Meanwhile, we confirmed by QPCR that differential expression analysis results (Figure 5e–l). These findings corroborate our in vitro observations.

**Figure 6:**
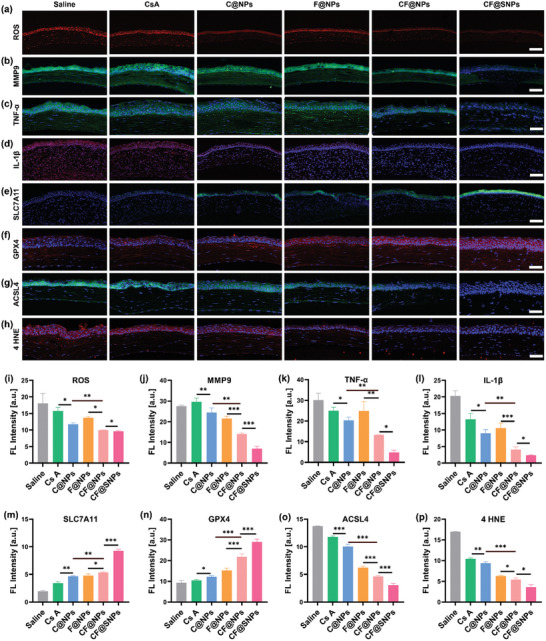
Anti‐ROS, anti‐ferroptosis, and anti‐inflammatory effects of CF@SNPs in DED mice were evaluated. Fluorescent images depict a) ROS levels (red), b) MMP‐9 (green), c) TNF‐α (green), and d) IL‐1β (red), with nuclei stained by DAPI (blue), scale bar: 50 µm. Representative images showcase the expression of e) SLC7A11 (green), f) GPX4 (red), g) ACSL4 (green), and h) 4HNE (red), with nuclei stained by DAPI (blue), scale bar: 50 µm. Quantitative analysis of average fluorescence intensity for i) ROS, j) MMP‐9, k) TNF‐α, l) IL‐1β, m) SLC7A11, n) GPX4, o) ACSL4, and p) 4HNE was presented. Show data as mean ± SD. n = 3. ^*^ *P* < 0.05, ^**^ *P* < 0.01, ^***^ *P* < 0.001.

Building upon these preliminary insights, we further investigated parameters associated with ferroptosis and inflammation in corneal tissue from various treatment groups. Oxidative stress serves as a crucial link between DED pathology and ferroptosis. To evaluate ROS levels, we proceed with immunofluorescence analysis (**Figure** **6**a,i). ROS fluorescence markedly increased by establishing the DED model, reaching an intensity of 18.1 ± 2.98. Treatment with CsA, C@NPs, or F@NPs mitigated ROS levels to some extent. Administration of CF@NPs and CF@SNPs significantly reduced ROS fluorescence to 9.98 ± 0.0231 and 9.62 ± 0.221, respectively, surpassing the efficacy of other treatments.

We conducted immunofluorescence assays to assess the levels of ferroptosis‐associated proteins, including SLC7A11, GPX4, ACSL4, and 4‐HNE. Following the establishment of the DED model, there was a notable reduction in the fluorescence intensity of SLC7A11 (Figure 6e,m) and GPX4 (Figure 6f,n), inversely correlated with ferroptosis. Treatment with CsA, C@NPs, and F@NPs mitigated this decline in protein expression to varying degrees, with the C@NPs group exhibiting superior efficacy compared to CsA. CF@NPs and CF@SNPs treatments significantly increased the expression levels of SLC7A11 and GPX4 proteins. Among them, the fluorescence intensity of the CF@SNPs group can reach 5.36 ± 0.138 (SLC7A11), 21.9 ± 1.43 (GPX4), and the effect of the CF@NPs group is significantly better than CF@NPs group, which the fluorescence intensity of the group can increase to 9.28 ± 0.281 (SLC7A11) and 29.0 ± 1.32 (GPX4). In contrast, ACSL4 (Figure 6j,o) and 4‐HNE (Figure 6h,p) proteins, positively associated with ferroptosis, exhibited significantly elevated fluorescence in the saline group. CsA, C@NPs, and F@NPs treatments moderately decreased fluorescence intensity, with the F@NPs group demonstrating more substantial improvements, albeit differing notably from the CF@NPs group. Notably, the CF@NPs group reduced fluorescence intensity to around one‐third of the saline group, with the CF@SNPs group showing superior efficacy.

As for inflammation‐related markers, immunofluorescence staining targeting MMP9 (Figures 6b,j), TNF‐α (Figure 6c,k), and IL‐1β (Figure 6d,l) were conducted. Following DED induction, the saline group exhibited elevated fluorescence intensity for MMP9, TNF‐α, and IL‐1β. Treatment with CsA provided marginal alleviation of inflammation. Notably, groups treated with C@NPs and F@NPs demonstrated moderate improvements in inflammatory status. Furthermore, CF@NPs and CF@SNPs exhibited remarkable suppression of severe inflammatory reactions by DED. The fluorescence intensity of MMP9, TNF‐α, and IL‐1β in the CF@SNPs group notably reduced to approximately half that of the CF@NPs group.

## 3 Conclusion

DED represents a serious ocular ailment with a rising global incidence, often driven by inflammation. However, conventional anti‐inflammatory therapy was suboptimal due to the side effects.^[^ ^43^ ^]^ This unexpected efficacy primarily arises from stressors like dryness and high osmotic pressure by exacerbating the inflammatory response and triggering oxidative stress pathways, synergistically contributing to DED pathogenesis.^[^ ^44^, ^45^ ^]^ Labile iron and lipid peroxidation‐dependent cell death, known as ferroptosis, is a significant player in oxidative stress‐induced corneal injury, underscoring the need for innovative therapeutic strategies. Recently, ferroptosis was observed in the corneal epithelia cells from the desiccation stress mouse model, highlighting the potential regulatory role of ferroptosis in DED progression.^[^ ^21^ ^]^

Our experimental findings revealed that establishing a DED model in HCECs led to elevated ROS levels, inflammation, ferrous ion content, lipid peroxidation, and increased expressions of ferroptosis‐related genes and proteins. Treatment with Fer‐1 significantly ameliorated these effects, validating the induction of ferroptosis in the in vitro DED model. Moreover, we found that Fer‐1 may mitigate ferroptosis through the p53‐SLC7A11 signaling pathway, thus improving DED outcomes.

Hence, we developed mucin‐targeting liposomes encapsulating the ferroptosis inhibitor Fer‐1 and the anti‐inflammatory drug CsA, CF@SNPs, for prolonging ocular surface retention and recovering the oxidative stress‐induced corneal epithelia death, which offers a promising avenue for DED treatment. In DED mice models, CF@SNPs treatment demonstrated superior therapeutic effects and improvement in clinical DED indicators, including amelioration of corneal injury, restoration of lacrimal gland structure, and goblet cell proliferation. Although inflammation was a common characteristic in the population suffering from DED, ROS attack, increased expression of p53, and cell death‐related markers have also been observed in the ocular surface of DED patients.^[^ ^46^ ^]^ However, the underlying mechanism is still obscure. Our in vitro and in vivo results demonstrated that dry eye stress‐induced upregulation of p53 further triggered SLC7A1111‐GSH‐dependent corneal epithelia ferroptosis. Combined administration of CsA and Fer‐1 substantially recovered corneal damage and DED indicators, further clarifying the coordinated benefits of synergistically targeting inflammatory and ferroptosis pathways.

**Figure 7:**
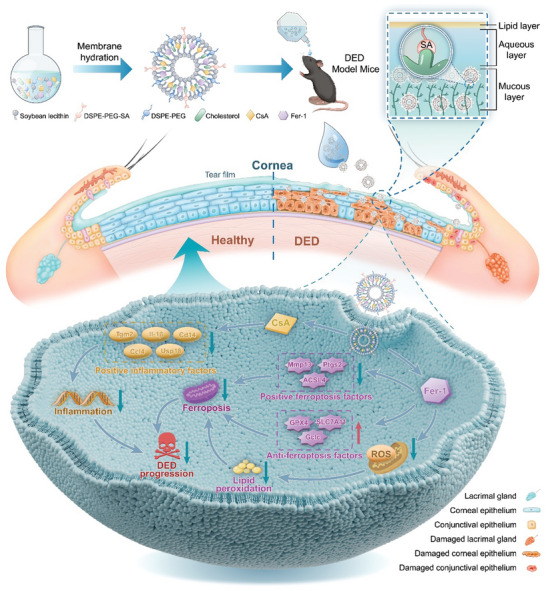
Schematic diagram showing the therapeutic effect and mechanism of CF@SNPs for treating DED.

In conclusion, CF@SNPs offer a promising solution for rescuing corneal damage in DED by disrupting the inflammatory cascade, ROS generation, and ferroptosis (**Figure** **7**). With demonstrated biological safety and ocular tolerance, CF@SNPs hold potential for future clinical applications in treating DED. Although ferroptosis is a new target for dry eye disease treatment, the in‐depth mechanism of CF@SNPs to treat dry eye disease via targeting ferroptosis needs further research.

## 4 Experimental Section

### Establishment of H_2_O_2_ Model In Vitro

HCECs were meticulously seeded into 6‐well plates at a predetermined density and allowed to increase for 24 h. Subsequently, they were subjected to pretreatment with 5 µM Fer‐1 (HY‐100579, MCE, China) or maintained in a complete culture medium for an additional 24 h. After this, the culture medium was substituted with an H_2_O_2_ (600 µM) serum‐free medium. Meanwhile, Erastin (HY‐15763, MCE, China) was employed as a positive control group. In this setting, HCECs were seeded in cell culture plates and allowed them to adhere overnight. They were pretreatment for 24 h with 5 µM Fer‐1 or complete cell culture medium before exposure to 10 µM Erastin.

### Establishment of HS Model In Vitro

HCECs were seeded onto tissue culture plates at varying densities and allowed to adhere for 24 h. Subsequently, they were treated with HS medium containing 500 mOsm L^−1^ NaCl, individually or in conjunction with 5 µM Fer‐1 for the specified duration.

### Cell Viability Assays

Cell survival rate was assessed via CCK‐8 and Live/Dead staining. For CCK‐8 assays, HCECs were seeded into 96‐well plates (1 × 10^4^ cells per well). Following a 24 h incubation period, the wells were allocated into seven groups: normal group (NC), Erastin, Erastin+Fer‐1, H_2_O_2_, H_2_O_2_+Fer‐1, HS, and HS+Fer‐1. HCECs were processed according to the model established previously. Subsequently, 100 µL of CCK‐8 solution (CK04, Dojindo, Japan) was added to each well, and absorbance was measured at 450 nm. Live/Dead staining followed a similar procedure in 24‐well plates (4 × 10^4^ cells per well) with the Live/Dead Viability/Cytotoxicity Kit (C2013S, Beyotime, China), incubated for 30 min at 37 °C. Subsequently, cells were washed thrice with PBS and visualized under a fluorescence microscope for image capture.

### Anti‐Oxidative Effects of Fer‐1

2 × 10^5^ HCECs were seeded per well into 12‐well plates. Following the previously outlined protocol, cells underwent treatment. After a 24 h incubation period, cells were rinsed and exposed to DCFH‐DA sourced from the ROS Assays Kit (S0033S, Beyotime, China). Subsequently, they were subjected to serum‐free DMEM/F12 for 37 °C, 30 min in darkness. Following triple PBS washes, the fluorescence microscope was to capture fluorescence images. Similarly, flow cytometry was used to determine the average fluorescence intensity of DCFH‐DA, and then the results were used to assess intracellular ROS generation in the collected cells. The resulting data were exported and analyzed by utilizing Flow‐Jo software.

### Intracellular MDA and GSH Assays

HCECs were seeded into 6‐well plates at a density of 4 × 10^5^ cells per well. Following the treatments as described earlier, cells were subjected to the Lipid Peroxidation MDA and GSH and GSSG Assays Kit (S0131S, S0053, Beyotime, China). Subsequent procedures were conducted according to the instructions provided with the reagent kit. Finally, a multifunctional enzyme marking instrument (SpectraMax iD5, MD) was employed to measure absorbance, allowing for calculating intracellular MDA and GSH content using formulas provided in the manuals.

### Intracellular Ferrous Ion (Fe^2+^) Assays

1 × 10^5^ HCECs were seeded per well into 12‐well plates and incubated them for 24 h. Following the established protocol, cells were treated accordingly. After three washes with PBS, Ferro Orange (F374, Dojindo, Japan) and Hoechst 33342 (C1022, Beyotime, China) dyes, prepared in serum‐free DMEM/F12 medium, were utilized for cell staining at 37 °C for 30 min. Subsequently, we directly used the fluorescence microscope to capture images.

### Evaluation of the Intracellular Lipo‐Peroxide Accumulation

HCECs were seeded onto 6‐well plates and subjected to various treatments following overnight incubation. After an additional 24 h of incubation, PBS washed twice cells, and the 10 µM C11‐BODIPY 581/591 probe (MP03792, Molecular Probes, USA) stained cells at 37 °C for 20 min in a tissue culture incubator. Subsequently, 500 µL fresh PBS three times resuspended cells and analyzed the results using flow cytometry.

### Anti‐Inflammatory Effect of Fer‐1 Evaluated by Immunocytochemistry

1 × 10^5^ HCECs were seeded per well onto 12‐well plates and were allowed to attach overnight. Subsequently, in vitro dry eye models were established. Immunocytochemistry staining of HCEC cells was performed using anti‐TNF‐α rabbit monoclonal antibody (1:100, ab215188, Abcam, UK), anti‐IL‐1β rabbit monoclonal antibody (1:50, ab254360, Abcam, UK), and anti‐MMP‐9 rabbit polyclonal antibody (1:50, ab283575, Abcam, UK). Following primary antibody incubation, goat anti‐Rabbit IgG H&L Alexa Fluor 488 (1:1000, ab150081, Abcam, UK) was utilized to detect protein levels.

### Preparation and Characterization of Nanoparticles

A thin‐film hydration method was utilized to fabricate liposomes. Initially, a lipid mixture comprising 1 mg of Fer‐1, 2.4 mg of Cyclosporin A, 8 mg of cholesterol (R‐H‐100001, Ruixi Biological Technology Co., China), 8 mg of 1,2‐distearoyl‐sn‐glycero‐3‐phosphoethanolamine‐N‐[methoxy‐(polyethylene glycol)‐2000] (DSPE‐PEG2000, R‐1028‐2K, Ruixi Biological Technology Co., China), and 2 mg of 1,2‐distearoyl‐sn‐glycero‐3‐phosphoethanolamine‐N‐[sialic acid(mucin)‐(polyethylene glycol)‐2000] (SA‐PEG2000‐DSPE, R‐G‐071, Ruixi Biological Technology Co., China), along with 30 mg of soybean lecithin (429415, Sigma–Aldrich, Germany), was dissolved in 10 mL of dichloromethane. The solution was then dried with a vacuum rotary evaporator under reduced pressure until a film formed on the bottom of the flask.^[^ ^22^ ^]^ The film was subsequently hydrated with 10 mL of double‐distilled water to generate a phospholipid film. The mixture were ultrasonicated at 4 °C for 2 min with 50 W power. CF@SNPs were obtained by centrifugation and filtration through a 0.22 µm‐filter. CF@NPs were synthesized by substituting 2 mg of DSPE‐PEG2000 with 2 mg of SA‐DSPE‐PEG2000 in the lipid mentioned above mixture. The same protocol was used to prepare hydrophobic fluorescent dye Nile Red‐NPs. DLS was used to determine the size and zeta potential of the liposomes. The morphology of CF@SNPs was characterized by a cryo‐TEM operated at an accelerating voltage of 200 Kv.^[^ ^22^ ^]^

### Precorneal Retention Evaluation

To assess the ocular surface retention time of liposomes, the differentiation in fluorescence intensity of the mice eyes was quantified using an IVIS Lumina imaging system at specific time points (0, 1, 5, 15, 30 min) with excitation/emission wavelengths of 535 nm/600 nm. To eliminate the influence of anesthetization, mice eyeballs were obtained without anesthesia, and frozen sections were prepared. For cryopreserved section imaging, equivalent concentrations of Nile Red, N@NPs, and N@SNPs were applied to the corneal surfaces of conscious normal mice. Subsequently, mice were euthanized at predetermined time points (30 s, 2, 5, 10, 15 min) following administration. Carefully removed the eyeballs from the orbits, then frozen in an optimal cutting temperature compound (4583, SAKURA, China) on dry ice. Thin slices of 7 µm thickness were obtained using a cryostat and then sealed with DAPI. A fluorescence microscope was used to observe the resulting images.

### DED Model Mice

C57BL/6 mice (aged 6–8 weeks, male, weigh 20 ± 2 g) provided by the Zhejiang Academy of Medical Sciences were housed under standard conditions with a consistent temperature of 22 ± 1 °C, a regular light/dark cycle, and access to food and water. Benzalkonium chloride (BAK) was used to induce the experimental DED model in mice. Specifically, 5 µL of 0.2% BAK eye drops (w/v) were administered to each eye twice daily for seven consecutive days. After establishing the model, The mice were randomly divided into six groups. A total of 10 µL of one of the following solutions were topical instilled twice daily in each group: 0.9% saline (w/v), CsA (199 µM), C@NPs (199 µM CsA), F@NPs (98 µM Fer‐1), CF@NPs (199 µM CsA, 98 µM Fer‐1), or CF@SNPs (199 µM CsA, 98 µM Fer‐1).

### Therapeutic Efficacy Assessment

Clinical indicators of DED include fluorescein sodium staining score, tear volume estimated via Schirmer test, and TUBT. Pentobarbital sodium was intraperitoneally injected at 50 mg kg^−1^ in all mice and then stained them with fluorescein sodium staining. Topical administration of 1% sodium fluorescein (w/v) 2 µL was performed, then manually closed the mice eyes 2–3 times. Used the slit lamp with cobalt blue illumination to take corneal photographs and scored according to clinical scoring criteria. Assigned a score for quadrant ranging from 0 to 4 based on the following criteria: 0) no staining; 1) mild punctate staining, <30 spots; 2) punctate staining > 30 spots, but not diffuse; 3) diffuse staining but no positive plaques; 4) positive plaques.^[^ ^47^, ^48^ ^]^ Red‐impregnated cotton threads to asses tear volume (Jing Ming Tech Co., China). Cotton thread was placed at one‐third of the palpebra inferior conjunctiva for 15 s, and the length of the impregnated portion was measured using the built‐in ruler of the reagent kit. 1 µL of 1% sodium fluorescein (w/v) was instilled into the lower conjunctival sac of the right eye to determine TBUT. The time at which the first black spot under the cobalt blue lamp of the slit lamp imaging system was observed and recorded it as the TBUT. Ten right eyes in each group were tested, and calculated the average for analysis.

### Statistical Analysis

Present the data as the mean ± standard deviation (SD). GraphPad Prism to conduct statistical analyses. Two‐sided Student's t‐tests to assess differences between the two groups. A one‐way analysis of variance (ANOVA) was employed for comparisons among three or more groups, followed by post‐hoc tests. The *P*‐value of <0.05 was considered statistically significant.

### Ethics Statement

Our animal experiments have all complied with the Association for Research in Vision and Ophthalmology Statement, and the Guidelines for the Animal Care and Use Committee, Zhejiang University. The Animal Ethics Committee, the Second Affiliated Hospital, School of Medicine, Zhejiang University (Approval number: 2022–200) approved all animal experimental protocols.

## Conflict of Interest

The authors declare no conflict of interest.

## Supporting information



Supporting Information

## Data Availability

The data that support the findings of this study are available from the corresponding author upon reasonable request.
