# Supplementary material for: Corneal Mucin‐Targeting Liposome Nanoplatforms Enable Effective Treatment of Dry Eye Diseases by Integrated Regulation of Ferroptosis and Inflammation
Source: Adv Sci (Weinh). 2024 Nov 28;12(3):2411172. doi: 10.1002/advs.202411172 (PMC11744570; doi:10.1002/advs.202411172)
Supplement: Supplementary file 1 — Supporting Information [file ADVS-12-2411172-s001.docx]

Supporting Information

Corneal Mucin-Targeting Liposome Nanoplatforms Enable Effective Treatment of Dry Eye Diseases by Integrated Regulation of Ferroptosis and Inflammation

*Yin Zhang, Tinglian Zhou, Kai Wang, Chenqi Luo, Dan Chen, Zeen Lv, Haijie Han, ^*^ Ke Yao^*^*

**Experimental Section**

Biocompatibility Assessment *in vivo*.

Male mice, randomly allocated into six groups, underwent topical administration of 10 µL of 0.9% saline (w/v), Cyclosporine A (CsA, 199 µM), C@NPs (199 µM CsA), F@NPs (98 µM Fer-1), CF@NPs (199 µM CsA, 98 µM Fer-1), and CF@SNPs (199 µM CsA, 98 µM Fer-1) twice daily for seven consecutive days. On the seventh day, slit lamp examination and photography of fluorescein sodium staining under both bright field and cobalt blue lamps were performed on the right eye of each mouse group. After euthanasia *via* cervical dislocation, the eyeballs were excised and processed into H&E sections. Concurrently, major organs, namely the heart, liver, spleen, lung, and kidney, were swiftly removed and fixed in H&E solution for subsequent biocompatibility evaluation.

Cell Culture.

Human corneal epithelial cells (HCECs) were purchased from the American Type Culture Collection (ATCC). HCECs were meticulously cultivated within Dulbecco's modified Eagle's medium nutrient mixture F-12 (DMEM/F12, C11220500BT, Gibco, China), meticulously augmented with 10% fetal bovine serum (FBS, FBS00315-1, Aus GeneX, Australia) and 1% penicillin/streptomycin (15140-122, Gibco, USA). The cultivation milieu maintained a constant temperature of 37 °C in an environment with 95% air and 5% CO_2_ to ensure optimal growth conditions.

Biocompatibility Assessment *in vitro*.

Cell viability was assessed using the CCK-8 assay. HCECs or Raw 264.7 cells were seeded into 96-well plates at densities of 1 × 10^4^ or 2 × 10^4^ cells per well, respectively. After 24 hours, the cells were exposed to various concentrations of CF@SNPs in a serum-free medium for 24 hours. The proportion of viable cells was determined using the CCK-8 assay following the manufacturer's protocol (CK04, Dojindo, Japan).

For Live/Dead staining, HCECs or Raw 264.7 cells were cultured in 24-well plates overnight at a density of 4 × 10^4^ cells per well. Subsequently, the cells underwent the same treatment as in the CCK-8 assays. Live/Dead Viability/Cytotoxicity Kit (C2013S, Beyotime, China) was added and incubated for 30 minutes at 37 ℃. The cells were washed three times with PBS and imaged under a fluorescence microscope.

Quantitative Real-Time Polymerase Chain Reaction (qRT-PCR).

HCECs were seeded into 6-well plates at a density of 4 × 10^5^ cells per well and subsequently categorized into different experimental groups. Following incubation, 1 mL of TRIzol reagent (15596026, Thermo Fisher Scientific, USA) was added per well to extract the total RNA. The extracted RNA was quantified using a NanoDrop Spectrophotometer, followed by reverse transcription using the Prime Script RT reagent Kit (RR036A, Takara, Japan). For qRT-PCR, a CFX96 Real-Time System with SYBR Green Supermix (RR420A, Takara, Japan) was employed. Three biological replicates represented each experimental group. Gene expression levels were normalized to *GAPDH* mRNA, and the fold difference in the expression of target genes (*GPX4*, *FTH*, *SLC7A11*, *ACSL4*, *PTGS2*, *TNF-α*, *IL-1β*, *MMP 9*) was determined using the comparative cycle threshold method. Table S1 lists tested cytokines and sequences of forward and reverse primers.

Western Blot Analysis.

Whole-cell extracts were prepared by lysing cells using a Tissue or Cell Total Protein Extraction Kit (C510003, Sangon Biotech, China), and protein concentrations were quantified using the BCA method. Subsequently, 20 µg of resolved protein lysates were subjected to SDS-PAGE. Target proteins were detected using the following antibodies: anti-Glutathione Peroxidase 4 rabbit monoclonal antibody (GPX4, 1:1000, ab125066, Abcam, UK), anti-FACL4 rabbit monoclonal antibody (1:10000, ab155282, Abcam, UK), anti-xCT rabbit monoclonal antibody (1:1000, ab175186, Abcam, UK), anti-Cyclooxygenase 2 rabbit monoclonal antibody (ab179800, COX2, 1:1000, Abcam, UK), and anti-Ferritin rabbit monoclonal antibody (1:1000, ab75973, Abcam, UK). HRP-conjugated mouse monoclonal anti-GAPDH antibody (1:1000, ab9482, Abcam, UK) was also used as a loading control.

Histological Assessment.

Paraffin-embedded corneal and lacrimal gland tissues were sectioned into 4 µm thin slices using a paraffin slicer and subjected to H&E staining. We used the Periodic Acid-Schiff stain kit (G1280, Solarbio, China) and followed the manufacturer's instructions for PAS staining of paraffin sections. Images were captured using a standard upright digital light microscope.

ROS Assessment.

The OCT-embedded eyeballs were sliced into thin sections. Subsequently, the sections were washed three times with PBS and stained with Dihydroethidium (D7008, Sigma, Germany) at 37 ℃ for 30 minutes in the dark. After staining, the sections were counterstained with DAPI Fluoromount-GTM (36308ES11, Yeasen, China) for 20 minutes. Finally, images were captured using a fluorescence microscope.

Immunofluorescence Staining.

The frozen tissues were sectioned into 7 µm thin slices. Subsequently, immunofluorescence staining of the slices was conducted using the following antibodies: anti-4 Hydroxynonenal mouse monoclonal antibody (4HNE, 1:25, ab48506, Abcam, UK), anti-GPX4 mouse monoclonal antibody (1:400, 67763-lg, Proteintech, China), anti-IL-1 beta rabbit monoclonal antibody (1:50, ab254360, Abcam, UK), anti-FACL4 rabbit monoclonal antibody (1:100, ab155282, Abcam, UK), anti-xCT rabbit monoclonal antibody (1:500, ab307601, Abcam, UK), anti-TNF-α rabbit monoclonal antibody (1:100, ab215188, Abcam, UK), and anti-MMP-9 rabbit polyclonal antibody (1:50, ab283575, Abcam, UK). These antibodies were utilized to determine the protein levels through immunofluorescence staining.

**Table S1.** Primers used in quantitative RT-PCR

| Target | Forward Primer | Reverse Primer |
| --- | --- | --- |
| *h-GPX4* | GAGGCAAGACCGAAGTAAACTAC | CCGAACTGGTTACACGGGAA |
| *h-FTH* | CCCCCATTTGTGTGACTTCAT | GCCCGAGGCTTAGCTTTCATT |
| *h-SLC7A11* | GGCTCCATGAACGGTGGTGTG | GCTGGTAGAGGAGTGTGCTTGC |
| *h-ACSL4* | CATCCCTGGAGCAGATACTCT | TCACTTAGGATTTCCCTGGTCC |
| *h-PTGS2* | TGGTCTGGTGCCTGGTCTGATG | CCTGCTTGTCTGGAACAACTGCTC |
| *h-TNF-α* | CCTCTCTCTAATCAGCCCTCTG | GAGGACCTGGGAGTAGATGAG |
| *h-IL-1β* | CCACAGACCTTCCAGGAGAATG | GTGCAGTTCAGTGATCGTACAGG |
| *h-MMP 9* | GAACCAATCTCACCGACAGG | GCCACCCGAGTGTAACCATA |
| *h-GAPDH* | GTCTCCTCTGACTTCAACAGCG | ACCACCCTGTTGCTGTAGCCAA |
| *m-Mmp13* | CAGTTGACAGGCTCCGAGAAATG | CACATCAGGCACTCCACATCTTG |
| *m-Ptgs2* | GTGCCTGGTCTGATGATGTATGC | TGAGTCTGCTGGTTTGGAATAGTTG |
| *m-Gclc* | GCACATCTACCACGCAGTCAAG | ACATCGCCTCCATTCAGTAACAAC |
| *m-Tgm2* | AGCCGATGATGTGTACCTAGACTC | TTGATGAACTTGACAGAGCCTTGG |
| *m-Il1b* | TCGCAGCAGCACATCAACAAG | TCCACGGGAAAGACACAGGTAG |
| *m-Cd14* | TGGCTTGTTGCTGTTGCTTCTG | ACCAATCTGGCTTCGGATCTGAG |
| *m-Ccl4* | TGCTCGTGGCTGCCTTCTG | GAGGTGTAAGAGAAACAGCAGGAAG |
| *m-Usp18* | CGATGCTGCTCAACTCTACCTTAC | TCCTGCGTCCAGATGGTGAAC |
| *m-Gapdh* | TGAACCGCCGACCTATCCTTAC | GCACAAACACGAACCTCAAAGC |


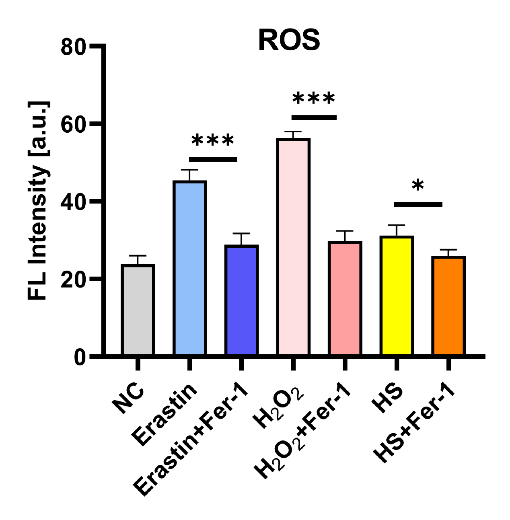


**Figure S1.** Quantitative results of ROS levels of HCECs after different treatments by DCFH-DA. Data are presented as mean ± SD; n = 3. ^*^*P* < 0.05; ^***^*P* < 0.001.


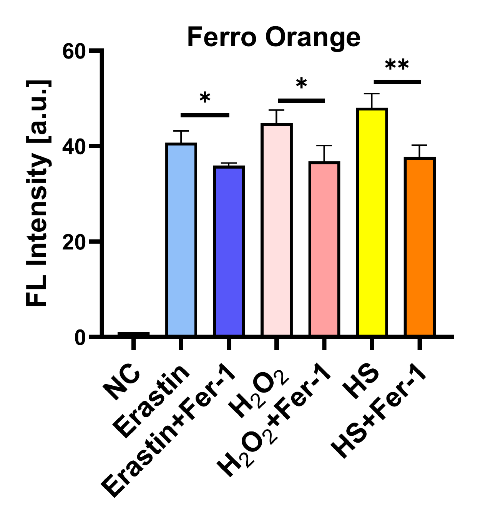


**Figure S2.** Quantitative results of Fe^2+^ levels of HCECs after different treatments by Ferro Orange. Data are presented as mean ± SD; n = 3. ^*^*P* < 0.05; ^**^*P* < 0.01.


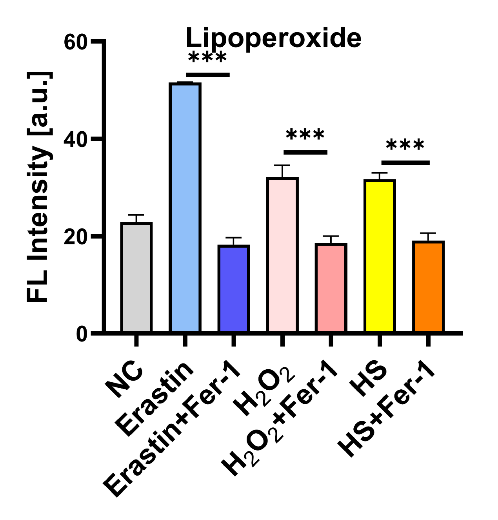


**Figure S3.** Quantitative results of lipid peroxidation levels of HCECs after different treatments by C11-BODIPY. Data are presented as mean ± SD; n = 3. ^***^*P* < 0.001.


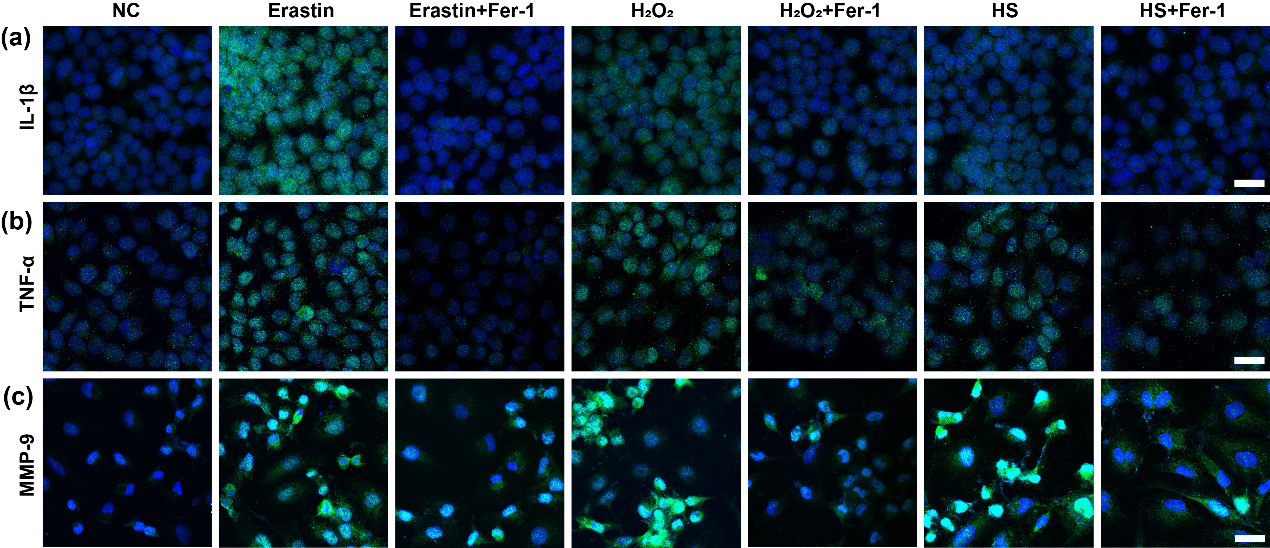


**Figure S4.** Anti-inflammation of Fer-1 in HCECs. Fluorescent images of a) IL-1β, b) TNF-α, and c) MMP-9. Nuclei are stained by DAPI (blue). The scar bar is 25 µm.


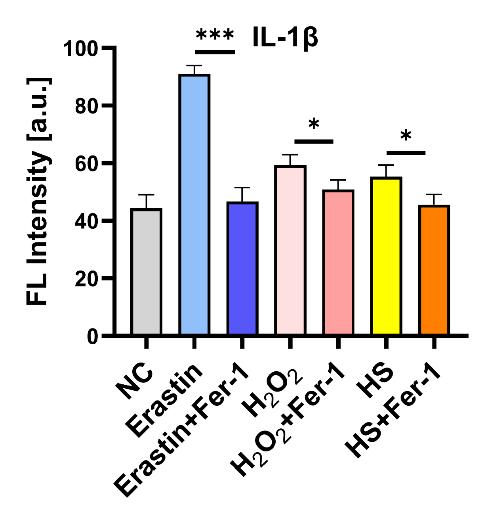


**Figure S5.** Quantitative analysis of average fluorescein intensity of IL-1β in HCECs. Data are presented as mean ± SD; n = 3. ^*^*P* < 0.05; ^***^*P* < 0.001.


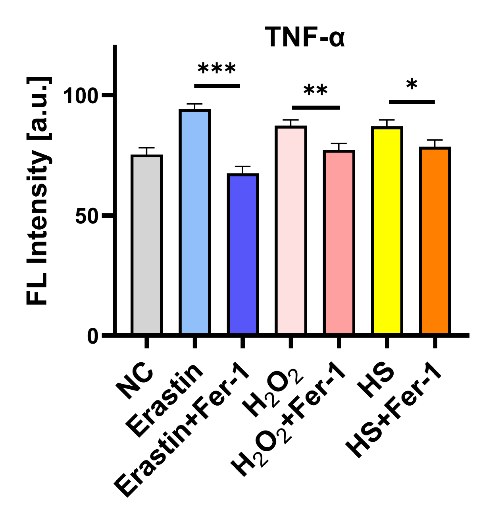


**Figure S6.** Quantitative analysis of average fluorescein intensity of TNF-α in HCECs. Data are presented as mean ± SD; n = 3. ^*^*P* < 0.05; ^**^*P* < 0.01; ^***^*P* < 0.001.


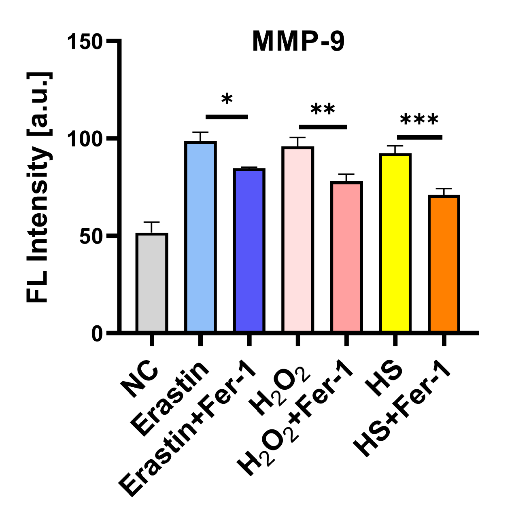


**Figure S7.** Quantitative analysis of average fluorescein intensity of MMP-9 in HCECs. Data are presented as mean ± SD; n = 3. ^*^*P* < 0.05; ^**^*P* < 0.01; ^***^*P* < 0.001.


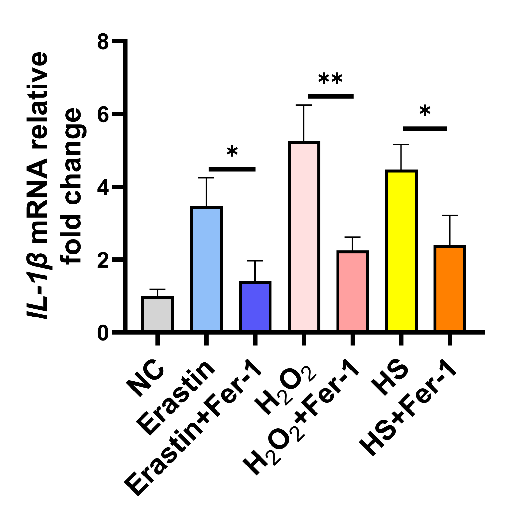


**Figure S8.** *IL-1β* mRNA relative fold change in HCECs after different treatments. Data are presented as mean ± SD; n = 3. ^**^*P* < 0.01.


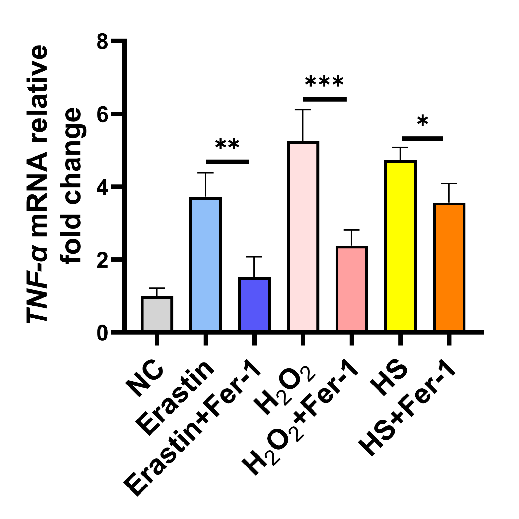


**Figure S9.** *TNF-α* mRNA relative fold change in HCECs after different treatments. Data are presented as mean ± SD; n = 3. ^**^*P* < 0.01.


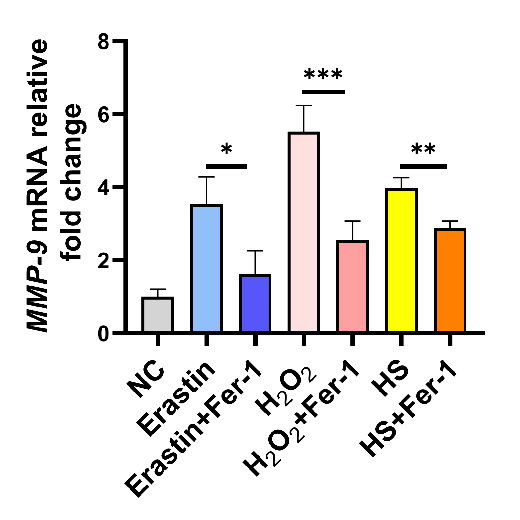


**Figure S10.** *MMP-9* mRNA relative fold change in HCECs after different treatments. Data are presented as mean ± SD; n = 3. ^**^*P* < 0.01.


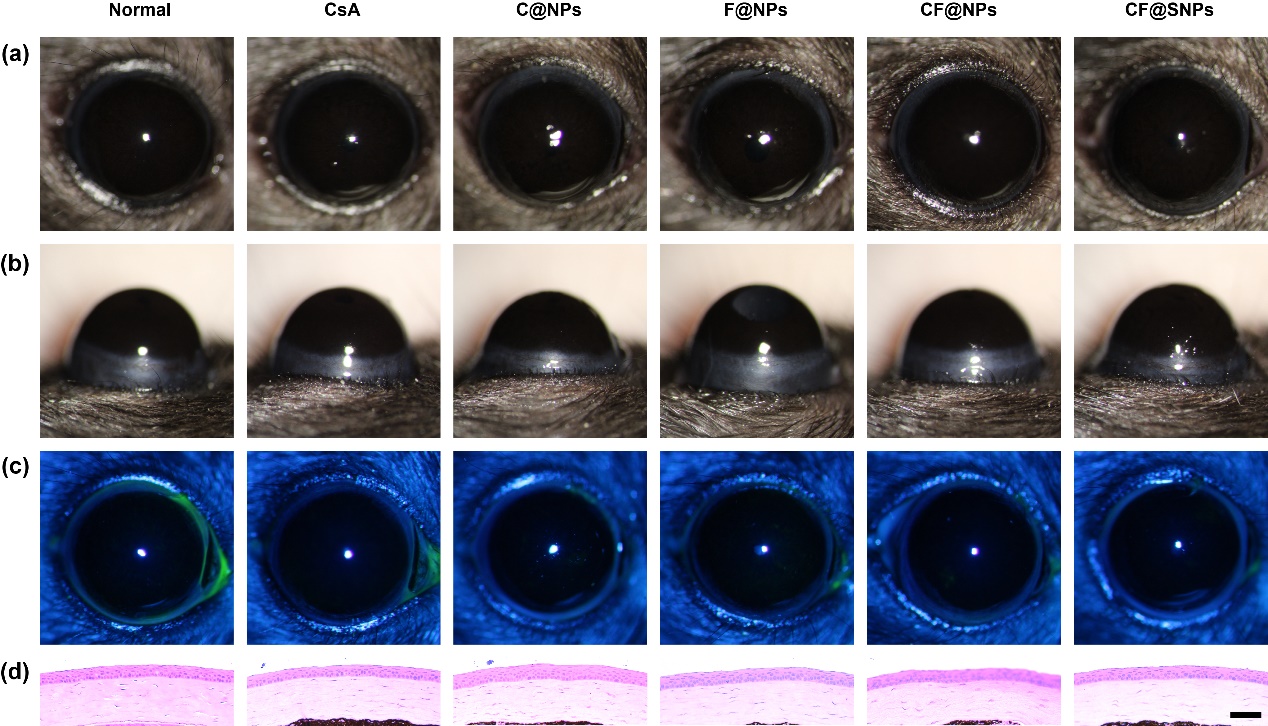


**Figure S11.** Safety assessment of various drugs on mouse corneas was conducted. Representative images of the a) anterior and b) lateral views of the mouse corneas were captured using a slit lamp in a bright field. c) Corneal fluorescein sodium staining images in mice following different drug interventions were obtained under a slit lamp with a cobalt blue filter. d) Representative images of corneal histology were acquired through H&E staining. The scale bar represents 100 µm.


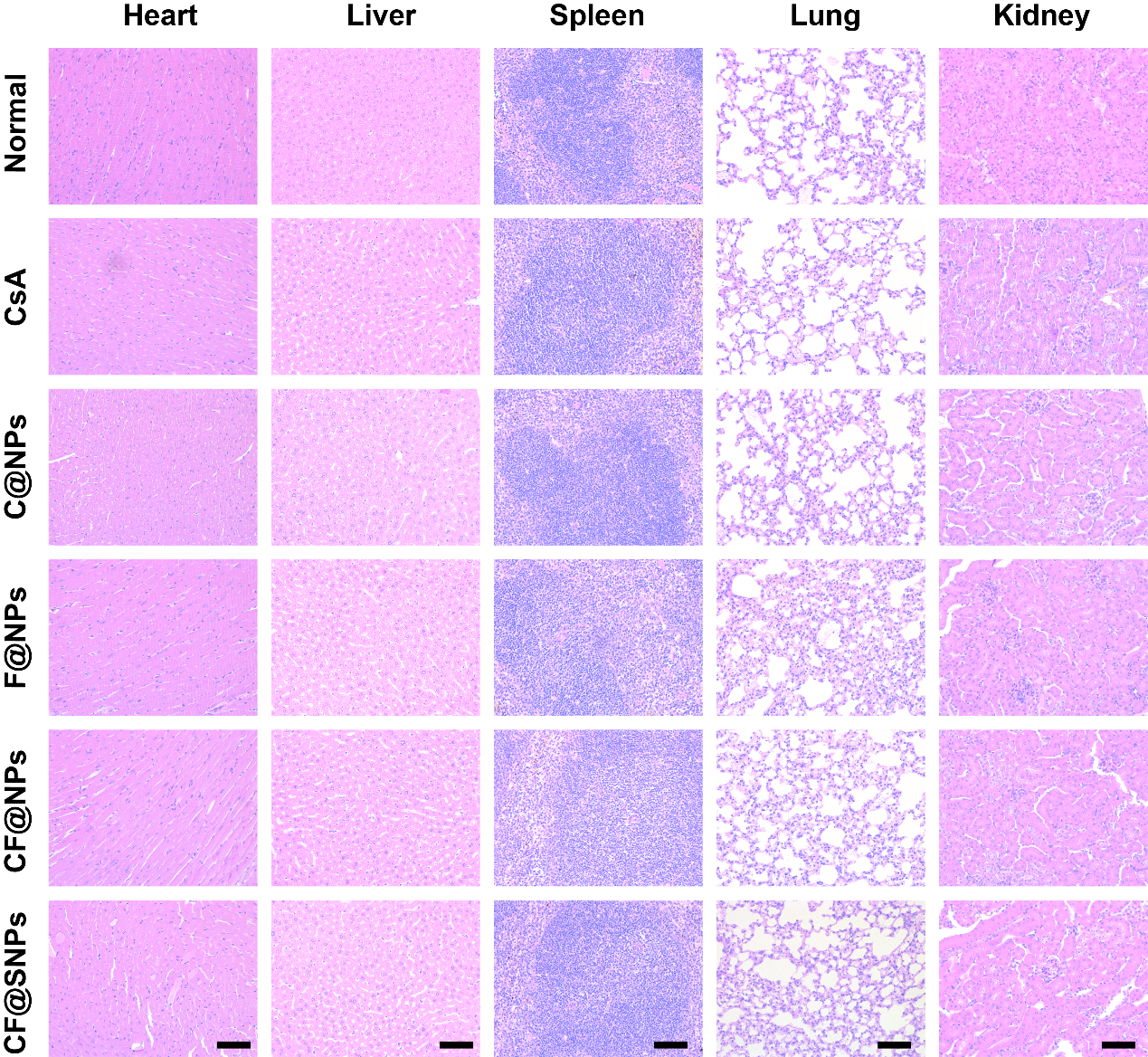


**Figure S12.** Safety assessment of various drugs on other organs in mice was performed. Representative images of the heart, liver, spleen, lung, and kidney were obtained through H&E staining. The scale bar represents 100 µm.


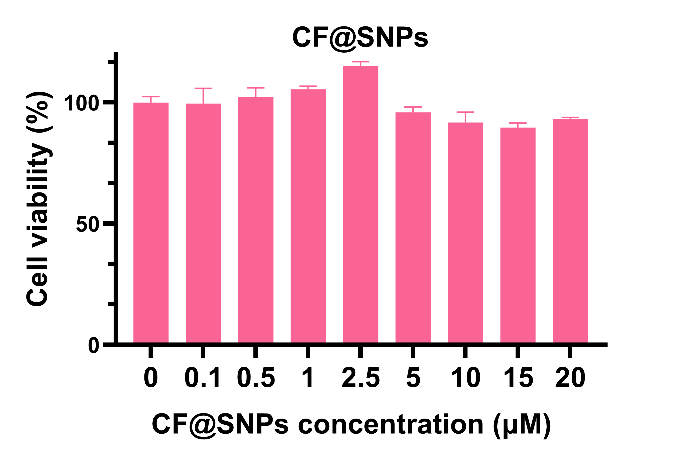


**Figure S13.** The cell viability of HCECs was assessed using the CCK-8 assay. Data are presented as mean ± SD; n = 5.


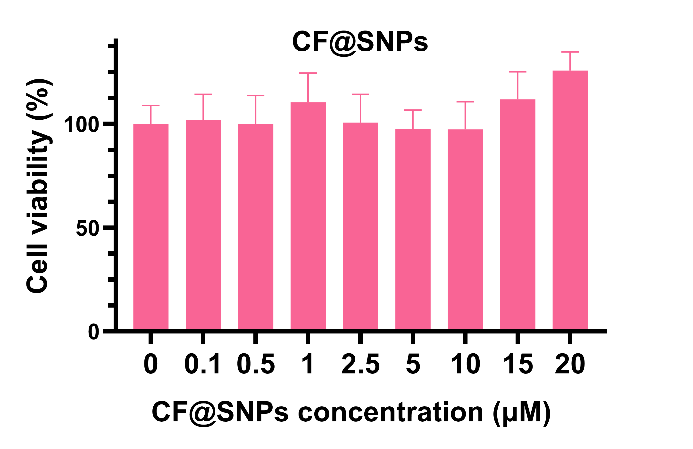


**Figure S14.** Cell viability of Raw 264.7 cells was assessed using the CCK-8 assay. Data are presented as mean ± SD; n = 5.


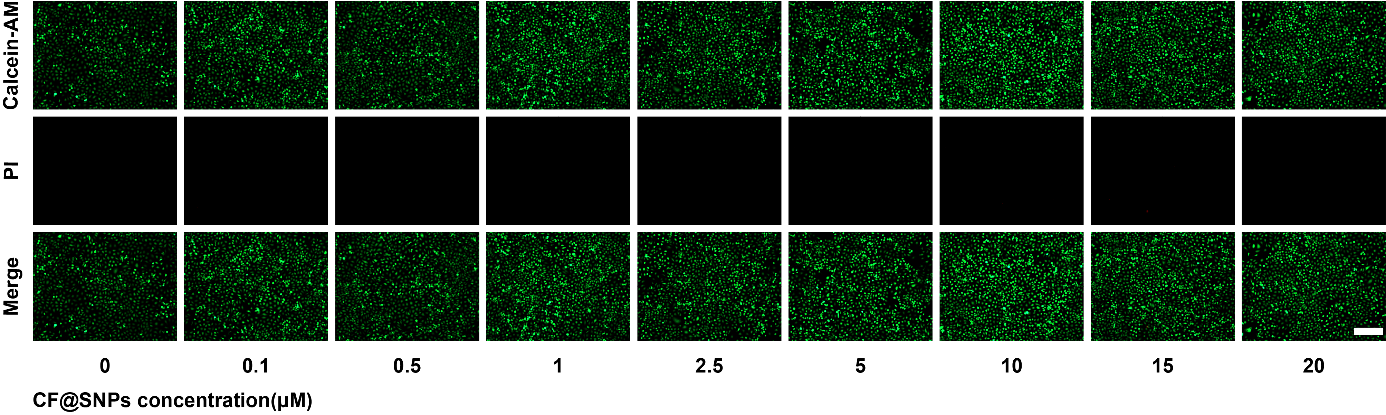


**Figure** **S15.** Representative Live/Dead images of different concentrations of CF@SNPs (Fer-1 was used as the concentration standard) in HCECs. Green: live cells; Red: dead cells. The scar bar represents 100 µm.


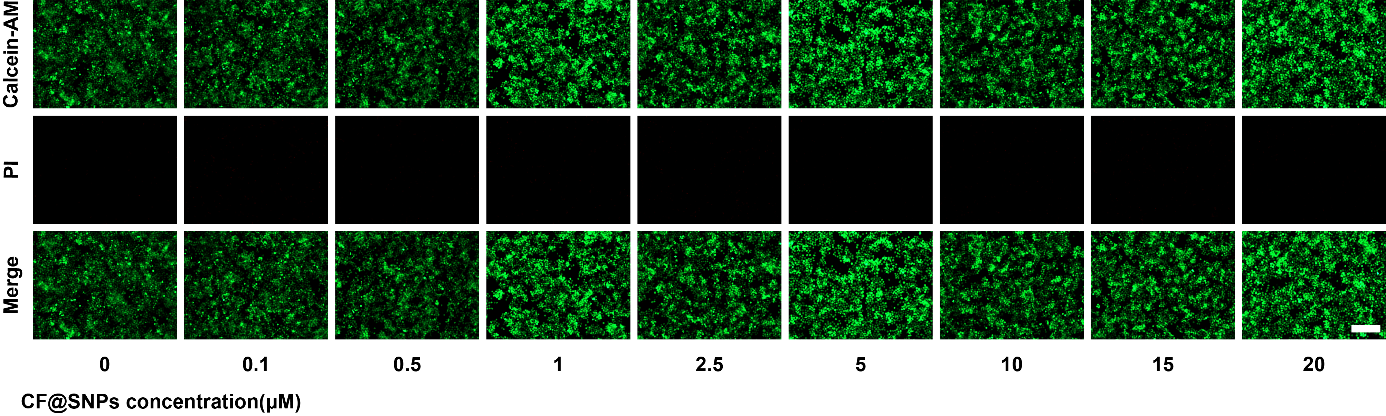


**Figure S16.** Representative Live/Dead images of different concentrations of CF@SNPs (Fer-1 was used as the concentration standard) in Raw 264.7 cells. Green: live cells; Red: dead cells. The scar bar represents 100 µm.


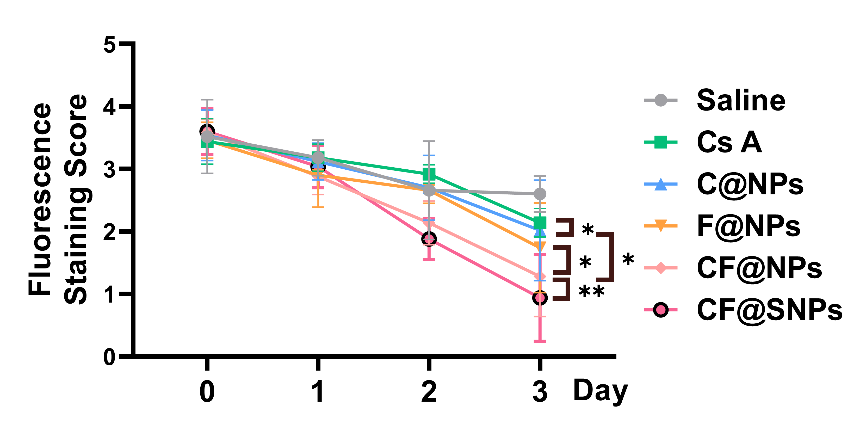


**Figure S17.** Scoring of corneal fluorescein sodium staining in mice with DED treated with different therapies was conducted on days 0, 1, 2, and 3. Data are presented as mean ± SD; n = 10. ^*^*P* < 0.05; ^**^*P* < 0.01.


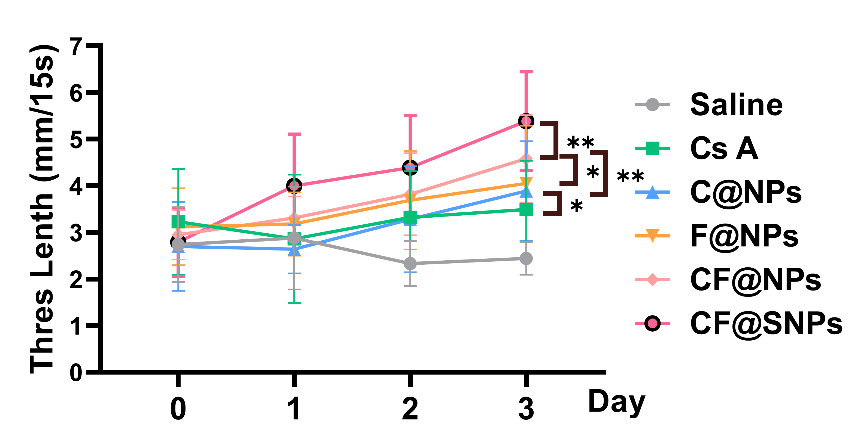


**Figure S18.** The phenol red cotton thread length in DED mice with different therapies was conducted on days 0, 1, 2, and 3. Data are presented as mean ± SD; n = 10. ^*^*P* < 0.05; ^**^*P* < 0.01.


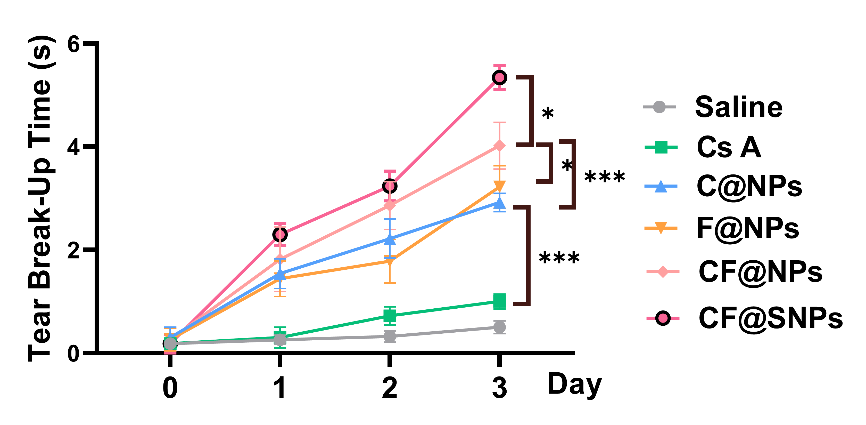


**Figure S19.** The tear break-up time in DED mice with different therapies was assessed on days 0, 1, 2, and 3. Data are presented as mean ± SD; n = 10. ^*^*P* < 0.05; ^**^*P* < 0.01; ^***^*P* < 0.001.


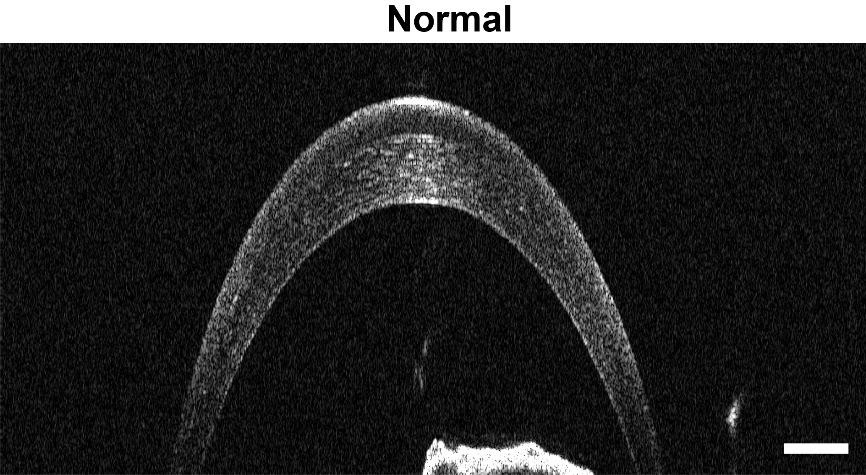


**Figure S20.** Anterior segment OCT images of normal mice. The scar bar represents 100 µm.
